# Particulate Matter-Induced Lung Inflammation Increases Systemic Levels of PAI-1 and Activates Coagulation Through Distinct Mechanisms

**DOI:** 10.1371/journal.pone.0018525

**Published:** 2011-04-11

**Authors:** G. R. Scott Budinger, Joanne L. McKell, Daniela Urich, Nancy Foiles, Ivy Weiss, Sergio E. Chiarella, Angel Gonzalez, Saul Soberanes, Andrew J. Ghio, Recep Nigdelioglu, Ece A. Mutlu, Kathryn A. Radigan, David Green, Hau C. Kwaan, Gökhan M. Mutlu

**Affiliations:** 1 Division of Pulmonary and Critical Care Medicine, Northwestern University Feinberg School of Medicine, Chicago, Illinois, United States of America; 2 Division of Hematology and Oncology, Northwestern University Feinberg School of Medicine, Chicago, Illinois, United States of America; 3 United States Environmental Protection Agency, Research Triangle Park, North Carolina, United States of America; 4 Section of Gastroenterology and Nutrition, Rush University Medical College, Chicago, Illinois, United States of America; University of Giessen Lung Center, Germany

## Abstract

**Background:**

Exposure of human populations to ambient particulate matter (PM) air pollution significantly contributes to the mortality attributable to ischemic cardiovascular events. We reported that mice treated with intratracheally instilled PM develop a prothrombotic state that requires the release of IL-6 by alveolar macrophages. We sought to determine whether exposure of mice to PM increases the levels of PAI-1, a major regulator of thrombolysis, via a similar or distinct mechanism.

**Methods and Principal Findings:**

Adult, male C57BL/6 and IL-6 knock out (IL-6^−/−^) mice were exposed to either concentrated ambient PM less than 2.5 µm (CAPs) or filtered air 8 hours daily for 3 days or were exposed to either urban particulate matter or PBS via intratracheal instillation and examined 24 hours later. Exposure to CAPs or urban PM resulted in the IL-6 dependent activation of coagulation in the lung and systemically. PAI-1 mRNA and protein levels were higher in the lung and adipose tissue of mice treated with CAPs or PM compared with filtered air or PBS controls. The increase in PAI-1 was similar in wild-type and IL-6^−/−^ mice but was absent in mice treated with etanercept, a TNF-α inhibitor. Treatment with etanercept did not prevent the PM-induced tendency toward thrombus formation.

**Conclusions:**

Mice exposed to inhaled PM exhibited a TNF-α-dependent increase in PAI-1 and an IL-6-dependent activation of coagulation. These results suggest that multiple mechanisms link PM-induced lung inflammation with the development of a prothrombotic state.

## Introduction

There is a well-defined link between acute exposure to ambient particulate matter (PM) and morbidity and mortality from ischemic cardiovascular events including acute myocardial infarction and ischemic stroke [1]. In the past several decades, the institution of pollution control measures has led to significant reductions in mean and peak PM levels in most parts of the developed world [1]. Nevertheless, in more recent population-based cohorts, investigators have observed a persistent contribution of PM to mortality from ischemic cardiovascular events [2]. The mechanisms by which PM exposure increases the risk of ischemic cardiovascular events are incompletely understood. Several groups of investigators have reported that humans and rodents exposed to PM develop an enhanced tendency toward thrombosis and impaired fibrinolysis in the first 24 hours after the exposure [3], [4], [5], [6], [7], [8], [9]. This time course is consistent with the lag time of PM-induced mortality reported in population based cohort studies [1].

Both humans and rodents exposed to PM develop pulmonary inflammation characterized by bronchoalveolar lavage (BAL) fluid pleocytosis accompanied by the local and systemic release of cytokines. Investigators examining the consequences of PM exposure in humans and rodents have consistently observed an increase in serum levels of IL-6 or its transcriptional target CRP [10], [11], [12], [13]. Consistent with these findings, we found that the release of IL-6 from alveolar macrophages was required for the development of a prothrombotic state in mice exposed to intratracheally instilled PM [14]. Following controlled exposures of men with stable coronary artery disease to diesel exhaust, Mills et al observed evidence of worsened myocardial ischemia accompanied by reduced endothelial release of plasminogen activator [15]. These results suggest that impaired fibrinolysis might also contribute to the observed increased risk in ischemic cardiovascular events in PM exposed populations. In this study, we sought to determine whether: (1) acute PM exposure via inhalation is sufficient to increase the systemic levels of PAI-1, the major regulator of fibrinolysis (2) whether exposure to PM via inhalation activates coagulation through the same mechanism we observed after exposure to intratracheally instilled PM and (3) whether similar mechanisms underlie both the induction of PAI-1 and the activation of coagulation following exposure to PM.

## Results

**Figure 1:**
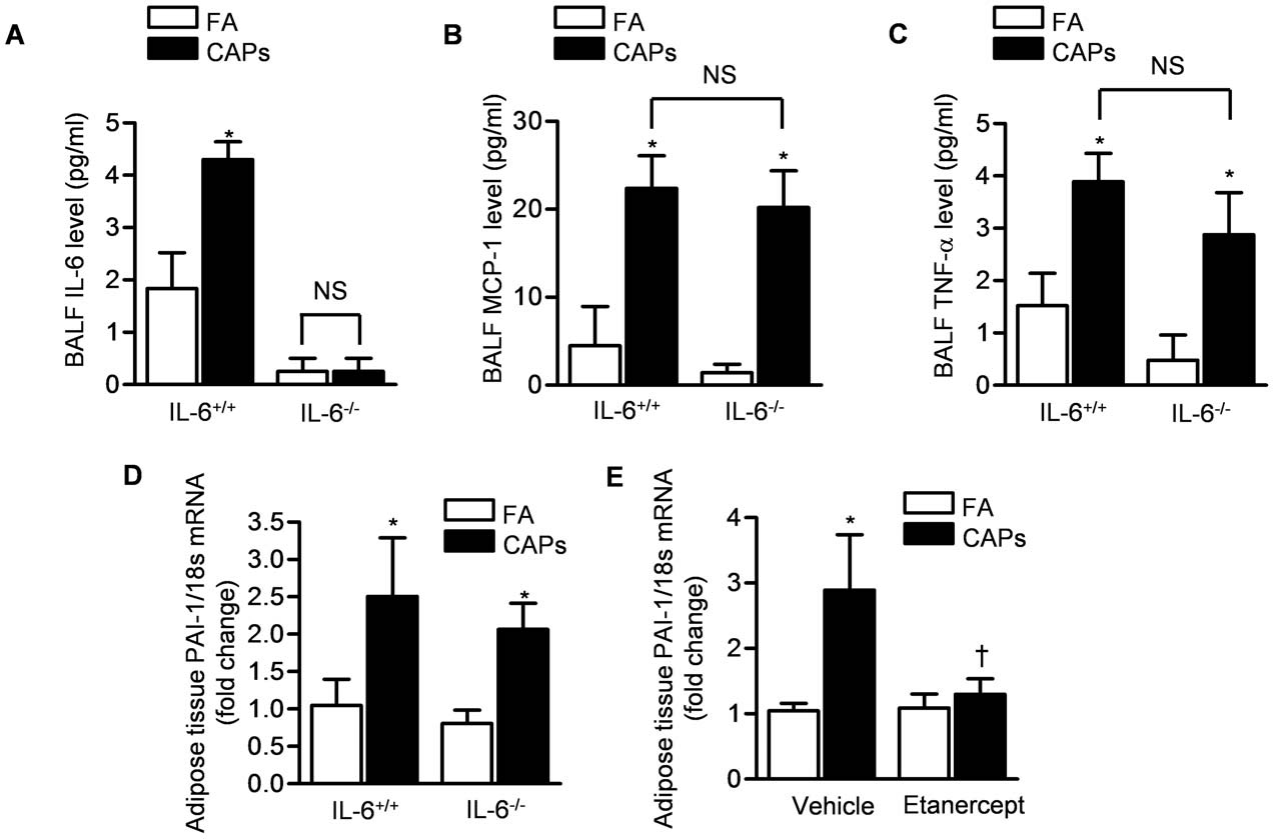
TNF-α is required for the increase in PAI-1 transcription induced by inhalational exposure to CAPs. Wild-type (IL-6^+/+^) and IL-6^−/−^ mice were exposed contemporaneously to CAPs (PM_2.5_) or filtered air (FA) for 8 hours daily on 3 consecutive days and BAL fluid was harvested at the end of the third day of exposure for measurement of Interleukin-6 (IL-6) (**A**), Monocyte Chemotactic Protein-1 (MCP-1) (**B**) and Tumor Necrosis Factor-α (TNF-α) (**C**). White adipose tissue was harvested from identically treated mice for measurement of the levels of PAI-1 mRNA (**D**). Wild type mice were treated with etanercept (10 mg/kg i.p.) or vehicle (saline) 3 days before and on the first day of exposure to CAPs or FA and white adipose tissue levels of PAI-1 mRNA were measured after the third day of exposure (**E**). (*p<0.05 CAPs vs. FA, n≥6/group).

PM_2.5_ increases white adipose tissue transcription of PAI-1 through a TNF-α-dependent mechanism. We exposed wild-type and IL-6^−/−^ mice to concentrated ambient PM_2.5_ (CAPs) or filtered air (FA) 8 hours per day for three days [16]. The mean daily ambient PM_2.5_ concentration was 12.7±3.1 µg/m^3^ during the study period while the mean concentration in the PM exposure chamber was 88.5±13.4 µg/m^3^. In mice exposed to CAPs, we found small but significant elevations in the levels of the pro-inflammatory cytokines IL-6, MCP-1 and TNF-α in the BAL fluid (Figure 1A–C). PAI-1 is the major systemic inhibitor of fibrinolysis and the white adipose tissue is a major site of PAI-1 production, particularly in response to inflammation [17], [18]. We exposed mice to CAPs 8 hours daily for three days and measured the levels of PAI-1 mRNA in the white adipose tissue. Compared with mice concomitantly exposed to filtered air, the CAPs exposed mice had a significant increase in white adipose tissue PAI-1 mRNA (Figure 1D). This increase in PAI-1 was similar in both wild-type and IL-6^−/−^ mice (Figure 1D). Canonical signaling through the TNF-α receptor activates the transcription factor NF-κB, which is a strong regulator of the PAI-1 promoter [19]. To test the hypothesis that the increase in PAI-1 transcription observed in CAPs exposed mice is regulated by TNF-α, we treated mice with the TNF-α inhibitor, etanercept (10 mg/kg, 3 days before and on the first day of exposure to CAPs). The administration of etanercept prevented the increase in white-adipose tissue PAI-1 mRNA in mice exposed to CAPs (Figure 1E).

**Figure 2:**
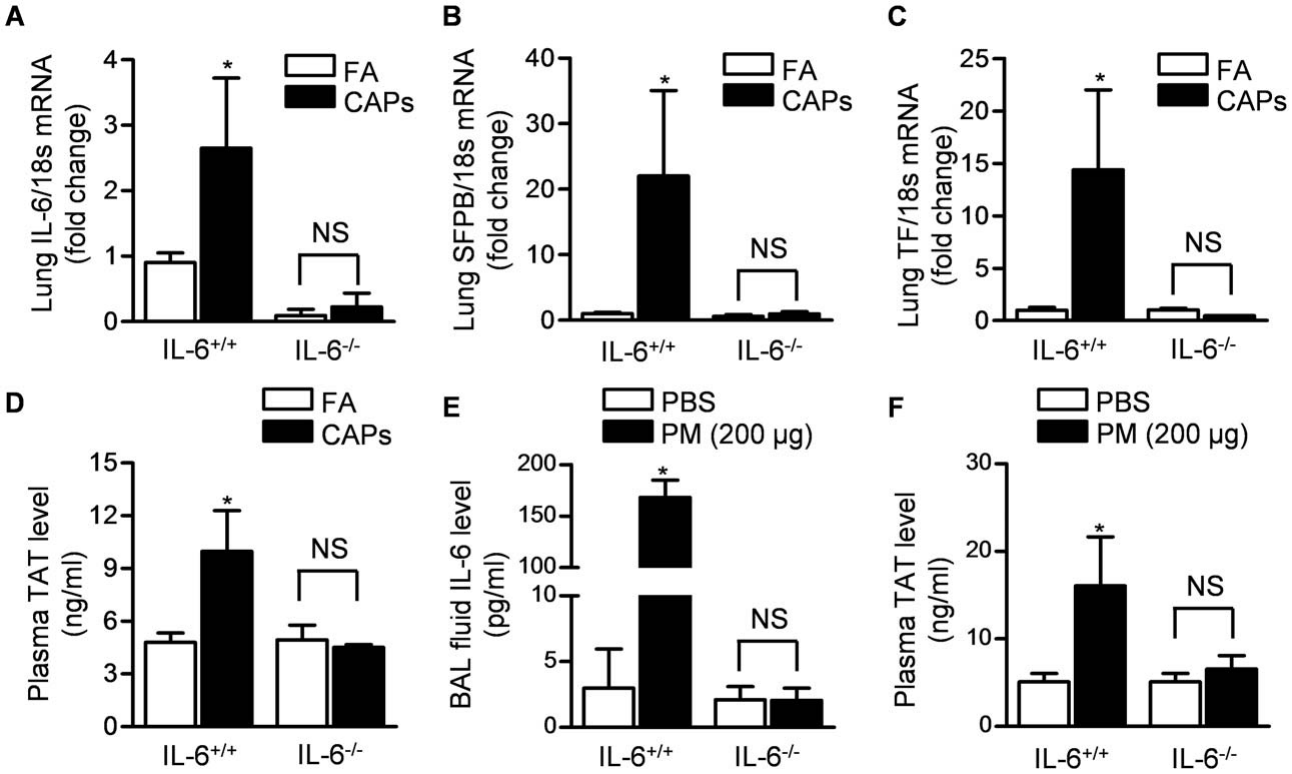
Interleukin-6 is required for activation of coagulation after inhalational exposure to concentrated ambient particles (CAPs) and the instillation of urban PM. Wild-type (IL-6^+/+^) and IL-6^−/−^ mice were exposed contemporaneously to CAPs (PM_2.5_) or filtered air (FA) for 8 hours daily on 3 consecutive days and lung tissue and BAL fluid were harvested at the end of the third day of exposure. Lung levels of mRNA encoding IL-6 and its transcriptional targets surfactant protein B (SFPB) and tissue factor (TF) (**A–C**) and plasma levels of thrombin antithrombin (TAT) complexes (**D**) were measured. Wild-type (IL-6^+/+^) and IL-6^−/−^ mice were treated intratracheally with urban PM (200 µg/mouse) and 24 hours later BAL fluid was obtained for measurement of IL-6 levels (ELISA) (**E**) and plasma was collected for measurement of thrombin-antithrombin complexes (**F**). (*p<0.05 CAPs vs. FA, n≥6/group).

Exposure to CAPs or instilled urban PM causes the IL-6-dependent activation of coagulation. Twenty-four hours after the intratracheal instillation of coarse PM, we reported that mice develop a prothrombotic state, which requires the release of IL-6 from alveolar macrophages [14]. We sought to determine whether this pathway is activated upon inhalation of PM_2.5_. We measured mRNA levels of IL-6 and its transcriptional targets surfactant protein B and tissue factor [20] in lung homogenates collected from mice exposed to CAPs or filtered air for 8 hours daily on three consecutive days. In wild-type mice, exposure to PM was associated with an increase in the transcription of IL-6, surfactant protein B (SFPB) and tissue factor (TF), while the levels of these transcripts did not increase in contemporaneously exposed IL-6^−/−^ mice (Figure 2A–C). Similarly, we found a significant increases in the levels of thrombin antithrombin complexes (TAT) in the plasma of wild-type but not IL-6^−/−^ mice exposed to CAPs (Figure 2D).

To determine whether the PM-induced changes in coagulation were similar after exposure to another urban particulate, we measured BAL fluid IL-6 levels and plasma TAT levels in mice following the intratracheal administration of urban PM collected by baghouse from Washington, DC [21]. When we treated wild-type mice intratracheally with increasing concentrations of this PM (10 µg, 100 µg and 200 µg), we observed a dose-dependent increase in the BAL fluid levels of IL-6 (Figure 2E and Figure S1A) and plasma TAT (Figure 2F and Figure S1B), which were not seen in IL-6^−/−^ mice.

### Exposure to urban PM causes the IL-6-dependent activation of coagulation and deposition of fibrin in the lung

**Figure 3:**
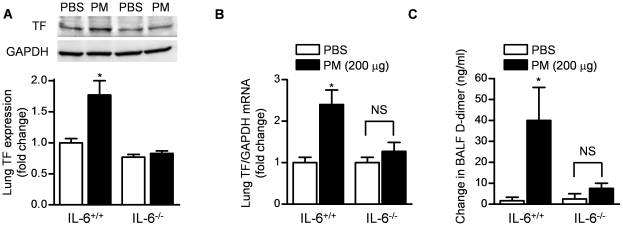
Urban PM induces the IL-6-dependent expression of tissue factor and deposition of fibrin in the lung. Wild-type (IL-6^+/+^) and IL-6^−/−^ mice were treated with PM (200 µg/mouse) or PBS for measurement of tissue factor (TF) protein (**A**) (immunoblotting and densitometry analysis shown) (n = 4/treatment) and mRNA (**B**) (qRT-PCR) in whole lung homogenates (n = 4/treatment). In identically exposed mice, BAL fluid fibrin was measured as the difference in the levels of D-Dimer pre- and post-digestion with exogenously administered plasmin (**C**). (*p<0.05, PM vs. PBS, n = 6/treatment).

Acute lung injury is associated with activation of coagulation and fibrin deposition locally in the lung both in humans and animals [22], [23]. To determine whether PM activates coagulation in the lung, we treated wild-type mice intratracheally with PM (200 µg) or PBS and examined lung homogenates for tissue factor mRNA (qRT-PCR) and protein (immunoblotting) 24 hours later. Compared to PBS treated mice, PM treated mice had significantly higher levels of tissue factor protein and mRNA (Figure 3A and 3B). This increase was not evident in IL-6^−/−^ mice. *In vitro*, we observed a dose-dependent increase in TF mRNA and protein expression in a lung epithelial cell line 24 hours after exposure to PM (Figure S2A and B). To determine whether this activation of coagulation locally in the lung is sufficient to cause an increase in fibrin, we measured the BAL fluid levels of fibrin by measuring the difference in D-dimer before and after treatment of the BAL fluid with exogenous plasmin as previously described [24]. Wild-type but not IL-6^−/−^ mice treated with intratracheal PM 24 hours earlier had significantly higher fibrin levels in the lung when compared to PBS treated animals (Figure 3C). We also observed increased staining for fibrin/fibrinogen in OCT embedded and snap frozen lung sections of PM compared with PBS treated mice (Figure S2C).

### The IL-6-mediated activation of coagulation is independent of lung inflammation

**Figure 4:**
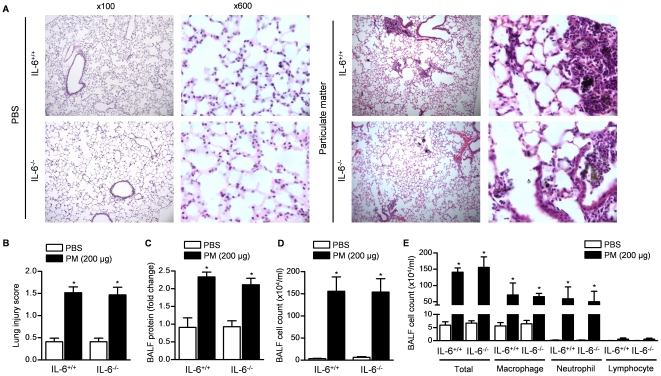
PM-induced lung injury is similar in wild-type and IL-6^**−/−**^ mice. Wild-type (IL-6^+/+^) and IL-6^−/−^ mice were treated with PM or PBS and harvested 24 hours later for histologic evaluation (**A**). Representative microphotographs of lung sections (hematoxylin and eosin stain, ×100 magnification, ×600 (inset) are shown. (**B**) Assessment of lung injury severity using a 5 point scoring system. (* p<0.05, PM vs. PBS, n≥4/group) Wild-type and IL-6^−/−^ mice were treated with urban PM (200 µg/mouse) or PBS and BAL fluid was obtained 24 hours later for measurement of (**C**) total protein (**D**) cell count and (**E**) differential cell count. (*p<0.05, PM vs. PBS, n = 8/group).

We treated wild-type and IL-6^−/−^ mice with increasing doses of PM and measured the severity of the resulting lung injury using a standardized scoring system [25]. The severity of the lung injury was similar in wild-type and IL-6^−/−^ mice (Figure 4A, B and Figure S3A, B). The intratracheal instillation of urban PM resulted in a dose-dependent increase in BAL fluid protein and an increase in BAL fluid macrophages and neutrophils in both wild-type and IL-6^−/−^ mice (Figure 4C–E and Figure S3C, D).

### Exposure to urban PM causes a TNF-α-dependent increase in PAI-1 in the lung and white adipose tissue

**Figure 5:**
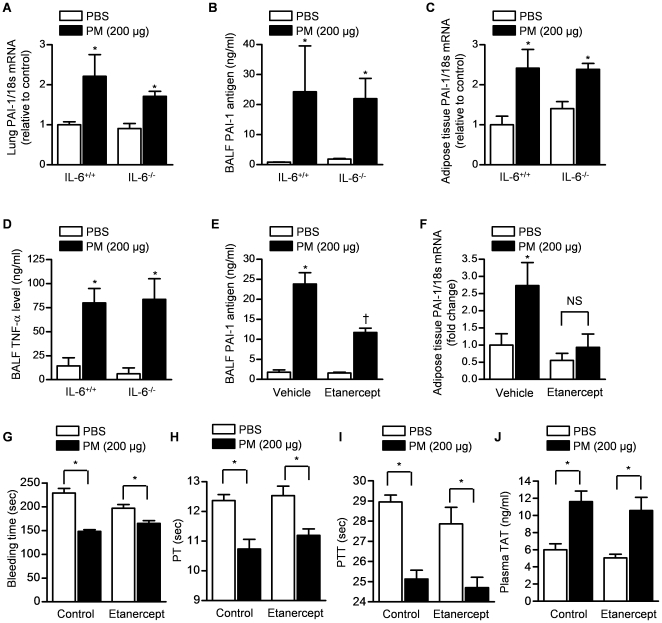
TNF-α but not IL-6 is required for urban PM-induced local and systemic release of PAI-1. Wild-type (IL-6^+/+^) and IL-6^−/−^ mice were treated with PM (200 µg/mouse) or PBS and lung and white adipose tissues, BAL fluid and plasma were harvested 24 hours later. (**A**) Whole lung PAI-1 mRNA, (**B**) BAL fluid PAI-1 antigen, (**C**) adipose tissue PAI-1 mRNA and (**D**) BAL fluid TNF-α levels were measured. Wild-type mice were treated with either etanercept, a TNF-α inhibitor (10 mg/kg i.p.) or vehicle (saline) 3 days and on the day of exposure to PM (200 µg/mouse) or PBS and BAL fluid, white adipose tissue and plasma were harvested or a bleeding time after tail vein cut performed 24 hours later. BAL fluid PAI-1 antigen (**E**), white adipose tissue PAI-1 mRNA (**F**), bleeding time after tail vein cut (**G**), plasma prothrombin time (PT) (**H**), partial thromboplastin time (PTT) (**I**) and plasma thrombin-antithrombin (TAT) complexes (**J**) were measured. (p<0.05, *PM vs. PBS, n≥4/group).

We measured lung PAI-1 mRNA, BAL fluid PAI-1 antigen levels and white adipose tissue PAI-1 mRNA in wild-type mice 24 hours after the intratracheal instillation of PM (200 µg/mouse) or PBS. Similar to the results observed in mice exposed to CAPs, these levels were higher in PM compared with PBS treated mice (Figure 5A–C). Significant elevations in PAI-1 were also observed in IL-6^−/−^ mice (Figure 5A–C). The PM-induced increases in TNF-α levels were similar in wild-type and IL-6^−/−^ mice (Figure 5D). Compared to control vehicle (PBS) treated animals, mice treated with etanercept (10 mg/kg i.p.) 3 days prior to and on the day of exposure to PM, the PM-induced increase in BAL fluid levels of PAI-1 (Figure 5E) and PAI-1 mRNA in adipose tissue (Figure 5F) were significantly reduced.

To determine whether the PM-induced increase in PAI-1 contributed to the increased tendency toward thrombosis induced by PM exposure, we treated wild type mice with vehicle or etanercept 3 days before and the day of the administration of intratracheal PM. Similar to our previous results using coarse PM, we observed a reduction in the PT, PTT, bleeding time and an increase in plasma TAT in vehicle treated mice 24 hours after the administration of PM (Figure 5G–J). The administration of etanercept did not prevent the PM-induced reduction in bleeding time, PT or PTT or increase in plasma TAT (Figure 5G–J).

## Discussion

We found that PM-induced inflammation is sufficient to induce a prothrombotic state and impair fibrinolysis in mice. Distinct mechanisms underlie the activation of coagulation and the inhibition of fibrinolysis as the activation of coagulation required the generation of IL-6, whereas the increase in PAI-1 required signaling through TNF-α independent of IL-6. In a standard and physiologically relevant inhalational model of PM exposure, we found evidence of IL-6-mediated gene transcription in the lung and the development of an IL-6-dependent prothrombotic state. In the same model, the administration of an inhibitor antibody against TNF-α prevented the systemic increase in PAI-1. The inhalation of PM was sufficient to increase expression of tissue factor, a transcriptional target of IL-6, and the intratracheal administration of PM was sufficient to induce the IL-6 dependent intrapulmonary deposition of fibrin.

Healthy people and people with pre-existing coronary artery disease exhibit a reduction in plasma tissue plasminogen activator in the first 24 hours after exposure to PM, suggesting that PM exposure might impair fibrinolysis. [4], [15], [26]. Fibrinolysis describes the degradation of fibrin by plasmin resulting in the generation of fibrin degradation products. Two fibrinolytic enzymes, urokinase-type plasminogen activator and tissue-type plasminogen activator are primarily responsible for the activation of plasmin from plasminogen [17]. Both of these enzymes are inhibited by PAI-1. Elevated levels of circulating PAI-1 have been identified as an independent risk factor for the development of ischemic cardiovascular events [27], [28] and have been associated with obesity, insulin resistance, aging, stress, inflammation and atherosclerosis [29]. We found that exposure to PM increases PAI-1 levels in the lung and in the white adipose tissue, the major source of circulating PAI-1 [18], [30].

The PAI-I promoter contains several functional binding sites for NF-κB [31], which is strongly activated by TNF-α [19] but lacks binding sites for STAT3, the primary transcription factor activated by IL-6 [32]. Consistent with these observations, we observed a similar PM-induced increase in PAI-1 in IL-6^−/−^ and wild-type animals. Etanercept is a fusion protein combining the human TNF receptor 2 with human IgG and has been shown to inhibit murine as well as human TNF-α signaling [33]. The administration of etanercept prior to PM exposure prevented the increase in PAI-1 transcription in the white adipose tissue and the increase in BAL PAI-1 induced by PM. Our results suggest that the inhibition of fibrinolysis is not required for the PM-induced tendency toward developing thrombosis as treatment with etanercept did not prevent the PM-induced reduction in PT, PTT or bleeding time or the increase in plasma TAT. While an increase in PAI-1 is often presented as evidence of inhibited fibrinolysis, we did not directly assess the blood or tissue fibrinolytic activity after PM exposure [29].

While our data do not address the source of TNF-α required for the induction of PAI-1 expression in the white adipose tissue, we previously reported that the intratracheal instillation of coarse PM was associated with only very small increases in the plasma levels of TNF-α [14]. Even this low level of TNF-α signaling, if sustained, may be sufficient to stimulate the production of PAI-1 by adipocytes. Alternatively, some particles may gain access to the circulation and induce a local inflammatory response in or near the adipose tissue.

The risk of ischemic cardiovascular events in humans increases with increasing exposure to PM [34], [35], [36]. While the mechanisms by which PM increases acute cardiovascular events are not fully understood, we and others have suggested that the consequences of PM-induced lung inflammation on hemostasis play an important role in this response [1], [3], [9], [11], [14], [15], [37], [38]. Exposure of humans to PM has been associated with increases in fibrinogen levels, shortening of the prothrombin time and the development of arterial (ischemic cardiovascular events) and venous (venous thromboembolism) thrombosis [1], [5], [37]. We previously reported that the intratracheal administration of coarse PM from Dusseldorf, Germany caused a transient prothrombotic state, which required the release of IL-6 from alveolar macrophages [14], [38]. Our current results extend these findings; we found that a fine PM fraction collected from Washington DC administered intratracheally and the inhalation of CAPs were both sufficient to cause an IL-6-dependent prothrombotic state.

Investigators have observed increases in both IL-6 and its transcriptional target C-reactive protein (expressed in humans but not mice) in humans following PM exposure [10], [11]. Our results highlight the importance of IL-6 in the development of the PM-induced prothrombotic state independent of other markers of inflammation. Following the intratracheal administration of PM, we found that the severity of the inflammatory response as measured by BAL fluid cell count, BAL fluid protein and histology were similar in wild-type and IL-6^−/−^ mice, however, the increase in TAT was not observed in the IL-6^−/−^ animals. In mice exposed to CAPs for 8 hours daily on 3 consecutive days, the increase in IL-6 was small but was still sufficient to increase transcription of the IL-6 target genes surfactant protein B and tissue factor and increase the plasma levels of TAT [20], [39]. The unique role of IL-6 in promoting thrombosis is well-described [39]. One mechanism, supported by our current results, is the transcriptional upregulation of tissue factor expression. Other important mechanisms include increased hepatic synthesis of the pro-coagulant factors fibrinogen, Factor VIII and vWF and reduced transcription of inhibitors of thrombosis including Protein C and Antithrombin. We previously reported that PM-treated animals exhibited selective, IL-6 dependent increases in fibrinogen, FVIII and vWF [14]. Investigators have not reported similar changes in coagulation in response to elevations in other pro-inflammatory cytokines [39].

We observed an IL-6-dependent increase in whole lung mRNA encoding tissue factor following exposure to CAPs and after the instillation of PM. We were only able to detect tissue factor protein in the lung and increased levels of fibrin after the instillation of relatively high doses of PM. While this may reflect the limited sensitivity of the available assays to detect the differential expression of these proteins in the lung, we cannot exclude the possibility that the increase in lung tissue factor results from the modest PM-induced lung injury that we have observed in the lungs of animals following PM exposure [7]. Our results are in agreement with those of Sun et al who observed an increase in TF expression in atherosclerotic plaques in a murine model of atherosclerosis after chronic exposure to CAPs [40]. Interestingly, increased levels of PAI-1 have been observed in atherosclerotic lesions and may be contribute to the development or progression of the disease [41].

We conclude that the lung inflammation induced by the inhalation of PM_2.5_ is sufficient to activate coagulation and inhibit fibrinolysis in the lung and systemically. PM induced IL-6 generation was required for the increased in tissue factor and fibrin in the lung and the systemic increase in TAT levels. By contrast, PM-induced TNF-α generation was required for the observed increase in PAI-1 but did not contribute to the PM-induced reduction in PT, PTT or bleeding time. Both mechanisms provide a link between exposure to PM and the observed increase in ischemic cardiovascular events in human populations.

## Materials and Methods

### Particulate Matter

For intratracheal exposure experiments in mice, we used an urban PM collected from ambient air in Washington, DC (National Institute of Standards and Technology Standard Reference Material, SRM 1649a). The characteristics of PM have been previously described [21]. For inhalational exposure, we used concentrated ambient PM_2.5_ (PM<2.5 µm) (CAPs) collected and concentrated via the Versatile Aerosol Concentration Enrichment System (VACES) [16].

### Animals and intratracheal administration of PM

The animal protocol (ASP-2009-1041) was approved by the Animal Care and Use Committee at Northwestern University. We used 8–12 week-old (∼25 g), male C57BL/6J mice (IL-6^+/+^) and IL-6 knockout mice (IL-6^−/−^) (Jackson Laboratories, Bar Harbor, Maine). We anesthetized the mice with isoflurane and intubated them orally with a 20-gauge angiocath [7], [14]. We instilled either PM suspended in 50 µl of sterile PBS (vortexed prior to instillation) or PBS (negative control). For some experiments, the administration of LPS (4 mg/kg, intratracheally) was used as a positive control [7], [14].

### Exposure of mice to CAPs

We exposed mice to CAPs (concentrated from ambient air in downtown Chicago with levels of traffic at ∼10× of ambient PM_2.5_ levels) for 8 hours per day (9 AM to 5 PM) for 3 days in one of two identical chambers connected to the outflow of a PM_2.5_ concentrator (VACES) [16], [42], [43]. We exposed control mice to filtered air in an identical chamber connected to the VACES in which a HEPA filter was placed on the inlet valve. We estimated ambient PM_2.5_ concentrations as the mean of reported values from the 3 EPA monitoring locations closest to our location. Particle counts in the chamber were measured with a TSI 3775 particle counter (Shoreview, MN) and used to determine the enrichment in the chamber compared with the ambient air as previously described [16], [42], [43].

### Collection of lung, bronchoalveolar lavage fluid and plasma

We removed the lungs and heart *en bloc*, inflated the lungs to 15 cm of H_2_O with 4% paraformaldehyde, embedded in paraffin and sectioned them (5-µm). We have previously described the procedures for harvesting of the lungs for immunoblotting and RNA isolation and the collection of BAL fluid and plasma [14], [44].

### Histology and immunohistochemistry

The severity of lung injury was scored by examining hematoxylin and eosin stained lung sections using a previously described 5-point lung injury scoring system (perivascular and peribronchial inflammation, hyaline membranes, alveolar and interstitial infiltrates, and alveolar hemorrhage) [25], [45].

### Quantification of lysed fibrin

We measured lysed, soluble fibrin in the BAL fluid as previously described [24]. We divided the BAL fluid into two equal aliquots; the first was snap frozen to determine pre-incubation D-dimer levels and the second was incubated with plasmin (0.32 AU/mL) at 37°C for 4 hours [24]. We measured D-dimer levels (ELISA, Diagnostica-Stago, Parsippany, NJ) in both aliquots and reported lysed fibrin levels as the difference between pre- and post-plasmin digestion D-dimer levels.

### Measurement of bleeding time, prothrombin time (PT), partial thromboplastin time (PTT), thrombin-antithrombin (TAT) complex and plasminogen activator inhibitor-1 (PAI-1) antigen

We measured PT, PTT, and plasma TAT levels as previously described [14] and PAI-1 antigen levels in BAL fluid by ELISA (Innovative Research, Novi, MI).

### Immunoblotting for tissue factor (TF)

For immunoblotting, we probed 10 µg of protein from mouse lung homogenates with a rabbit TF antibody (American Diagnostica, Stamford, CT, 1 µg/ml). We visualized the protein bands were using chemiluminescence and analyzed the resulting images with Image J software. We probed the same blots with and antibody against glyceraldehyde 3-phosphate dehydrogenase (GAPDH) (loading control).

### Quantitative real-time reverse transcription PCR (qRT-PCR) measurement of RNA

We isolated total RNA using a commercially available system (TRIzol, Invitrogen, Carlsbad, CA) from mouse lungs [44] and performed qRT-PCR reactions using IQ SYBR Green superscript with the primers listed below, analyzed on a Biorad IQ5 Real-Time PCR Detection System. We employed the Pfaffl method to analyze the normalized data as previously described [44]. PAI-1: (forward 5′-ACGCCTGGTGCTGGTGAATGC-3′, reverse 5′-ACGGTGCTGCCATCAGACTTGTG-3′), 18s: (forward: 5′-GGGTCGGGAGTGGGTAATT3′, reverse 5′-GAGAGGGAGCCTGAGAAAC-3′) [46] and TF: (forward 5′-CTACTGTTT CAGTGTTCAAGCAGTGA-3′, reverse 5′-CAGTGC AATATAGCATTTGCAGTAGC-3′) [47].

### Cell culture

A549 cells were obtained from the American Type Culture Collection (ATCC, Manassas, VA) and maintained in Dulbecco's modified Eagle's medium supplemented with L-glutamine (0.3 µg/ml), nonessential amino acids, penicillin (100 U/ml), streptomycin (200 µg/ml), and 10% fetal bovine serum (GIBCO, Grand Island, NY). For each experiment, we used a seeding density of 3.0×10^5^ cells/ml/well plated onto six-well plates (Costar, Cambridge, MA). The cells were grown to confluence over 24 h in a humidified 95% air/5% CO_2_ incubator at 37°C.

### Statistical analysis

We report all data as mean ± SEM. We subjected all data to a one-way ANOVA analysis. When ANOVA indicated a significant difference, we explored individual difference with the Student's t-test using Bonferroni correction for multiple comparisons (Prism 4, Graphpad, San Diego, CA). Statistical significance was defined as p<0.05.

## Supporting Information

Figure S1 **The intratracheal instillation of urban PM is associated with a dose-dependent increase in lung IL-6 and plasma thrombin antithrombin complexes.** Wild-type mice were treated with increasing doses of urban PM or vehicle (PBS) and BAL fluid and plasma were obtained 24 hours later. Mice treated with LPS (4 mg/kg, intratracheally) were used as a positive control. BAL fluid levels of IL-6 (**A**) and plasma levels of thrombin antithrombin (TAT) complexes (**B**) were measured. Each bar represents 4 or more animals; p<0.05, *PM compared with PBS control, †LPS vs. PM 200 µg/mouse.
(TIF)

Figure S2 **Exposure of lung epithelial cells to PM results in increased expression of tissue factor mRNA and protein and increased deposition of fibrin in the lung.** Lung epithelial cells (A549 cells) were treated with PBS or PM and processed, lysed 12 hours and 24 hours, respectively to measure levels of tissue factor mRNA (**A**) and protein (**B**). Protein is normalized to tubulin measurement by densitometry (ImageJ software); mRNA is normalized to 18S mRNA. Results are representative of three separate experiments. *p<0.05 for comparison between PM and PBS treatment groups. Mice were treated with increasing doses of urban PM or vehicle (PBS). LPS (4 mg/kg, intratracheally) was used as a positive control. OCT and snap frozen lung sections were obtained 24 hours after treatment and stained using an antibody that recognizes both fibrin and fibrinogen (**C**). Representative sections (400×) from 4 mice are shown.
(TIF)

Figure S3 **The intratracheal instillation of urban PM is associated with a dose-dependent increase in lung injury and inflammation.** Mice were treated with increasing doses of urban PM or vehicle (PBS) and lung tissue, BAL fluid and plasma were obtained 24 hours later. Mice treated with LPS (4 mg/kg, intratracheally) were used as a positive control. Representative lung sections from 3 mice treated with the indicated does (100× and 400×) are shown (**A**). The stained lung sections were scored using a previously described lung injury severity scoring system [25] for the presence of (1) perivascular and peribronchial inflammation (Inflammation), (2) hyaline membranes, (3) alveolar infiltrates, (4) interstitial infiltrates and (5) alveolar hemorrhage (**B**). BAL fluid was obtained for measurement of total protein (**C**) and cell count (**D**). Each bar represents 4 or more animals; p<0.05, *PM compared with PBS control, †LPS vs. PM 200 µg/mouse).
(TIF)
