# Supplementary figures and images for: Particulate Matter-Induced Lung Inflammation Increases Systemic Levels of PAI-1 and Activates Coagulation Through Distinct Mechanisms
Source: PLoS One. 2011 Apr 11;6(4):e18525. doi: 10.1371/journal.pone.0018525 (PMC3073968; doi:10.1371/journal.pone.0018525)

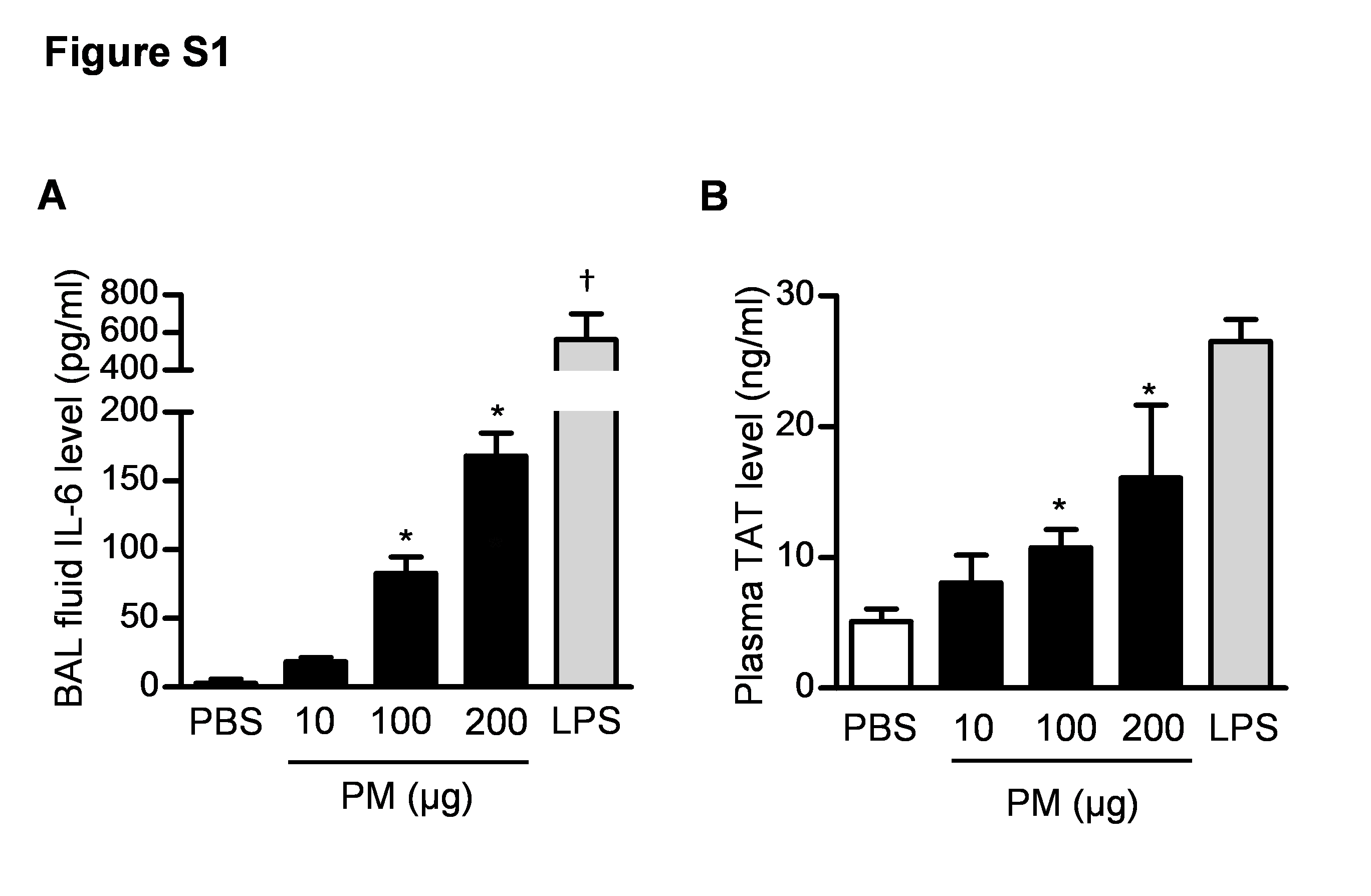

Supplement: Figure S1 — The intratracheal instillation of urban PM is associated with a dose-dependent increase in lung IL-6 and plasma thrombin antithrombin complexes. Wild-type mice were treated with increasing doses of urban PM or vehicle (PBS) and BAL fluid and plasma were obtained 24 hours later. Mice treated with LPS (4 mg/kg, intratracheally) were used as a positive control. BAL fluid levels of IL-6 (A) and plasma levels of thrombin antithrombin (TAT) complexes (B) were measured. Each bar represents 4 or more animals; p<0.05, *PM compared with PBS control, †LPS vs. PM 200 µg/mouse. (TIF) [file pone.0018525.s001.tif]

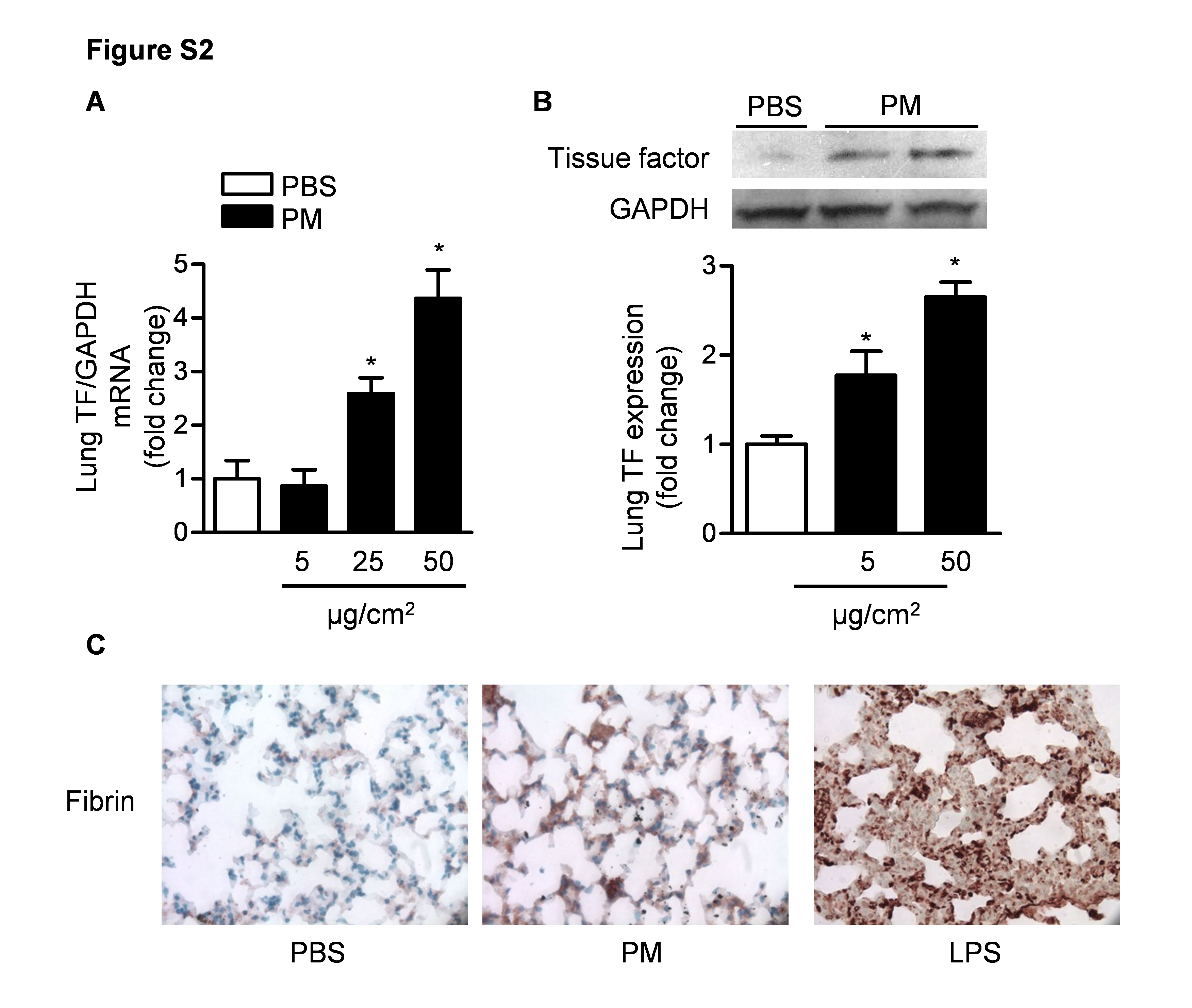

Supplement: Figure S2 — Exposure of lung epithelial cells to PM results in increased expression of tissue factor mRNA and protein and increased deposition of fibrin in the lung. Lung epithelial cells (A549 cells) were treated with PBS or PM and processed, lysed 12 hours and 24 hours, respectively to measure levels of tissue factor mRNA (A) and protein (B). Protein is normalized to tubulin measurement by densitometry (ImageJ software); mRNA is normalized to 18S mRNA. Results are representative of three separate experiments. *p<0.05 for comparison between PM and PBS treatment groups. Mice were treated with increasing doses of urban PM or vehicle (PBS). LPS (4 mg/kg, intratracheally) was used as a positive control. OCT and snap frozen lung sections were obtained 24 hours after treatment and stained using an antibody that recognizes both fibrin and fibrinogen (C). Representative sections (400×) from 4 mice are shown. (TIF) [file pone.0018525.s002.tif]

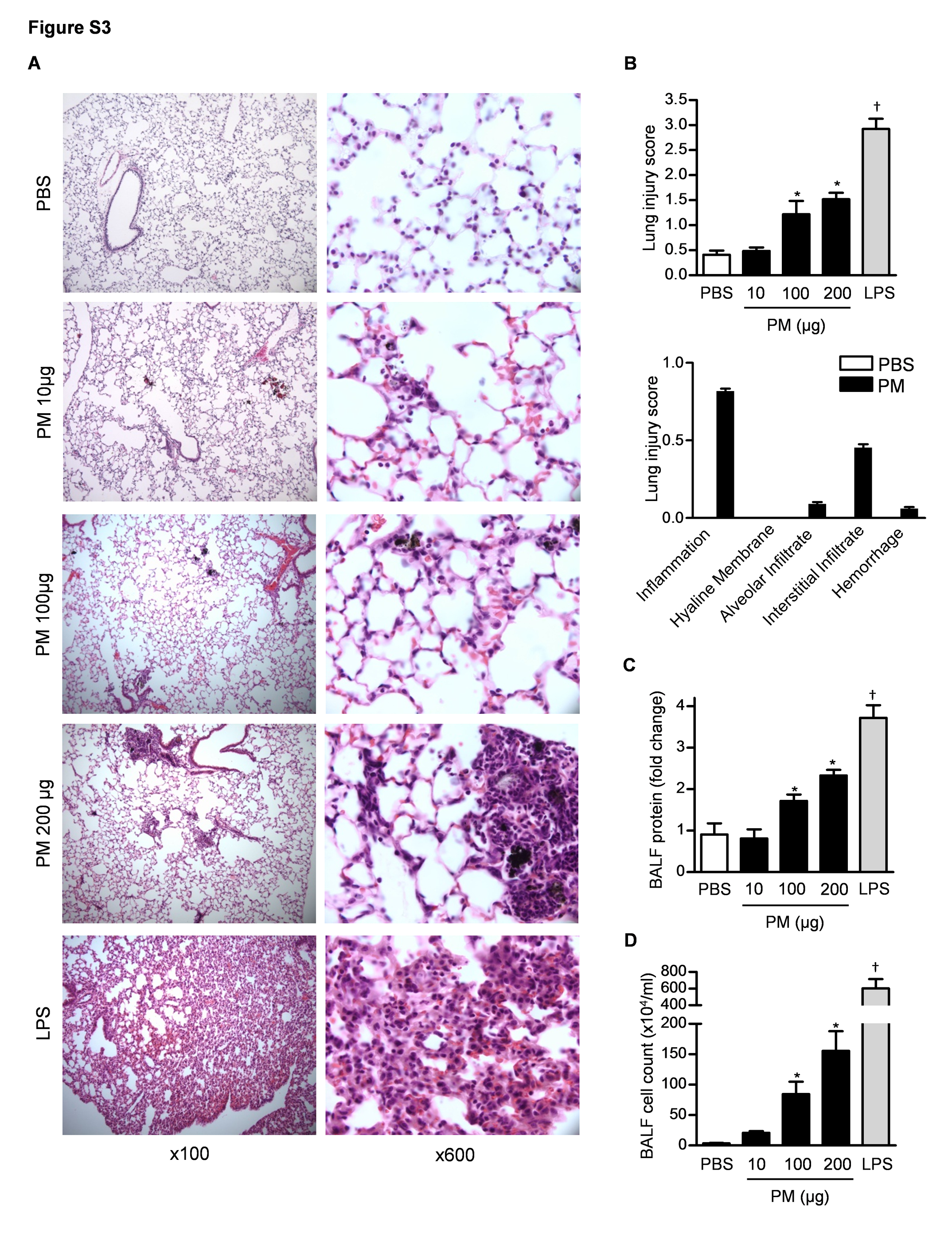

Supplement: Figure S3 — The intratracheal instillation of urban PM is associated with a dose-dependent increase in lung injury and inflammation. Mice were treated with increasing doses of urban PM or vehicle (PBS) and lung tissue, BAL fluid and plasma were obtained 24 hours later. Mice treated with LPS (4 mg/kg, intratracheally) were used as a positive control. Representative lung sections from 3 mice treated with the indicated does (100× and 400×) are shown (A). The stained lung sections were scored using a previously described lung injury severity scoring system [25] for the presence of (1) perivascular and peribronchial inflammation (Inflammation), (2) hyaline membranes, (3) alveolar infiltrates, (4) interstitial infiltrates and (5) alveolar hemorrhage (B). BAL fluid was obtained for measurement of total protein (C) and cell count (D). Each bar represents 4 or more animals; p<0.05, *PM compared with PBS control, †LPS vs. PM 200 µg/mouse). (TIF) [file pone.0018525.s003.tif]
